# Amplitude of the V1-R Wave Predicts Survival After Transcatheter Aortic Valve Replacement

**DOI:** 10.3390/medicina61091618

**Published:** 2025-09-08

**Authors:** Orhan Ince, Esra Donmez, Sevgi Ozcan, Kamil Gulsen, Murat Ziyrek, Emrah Ozdemir, Muhammed Furkan Deniz, Irfan Sahin, Ertugrul Okuyan

**Affiliations:** 1 Department of Cardiology, Bagcilar Training and Research Hospital, Istanbul 34200, Turkey; esra.donmez@sbu.edu.tr (E.D.); sevgi.ozcan@sbu.edu.tr (S.O.); drfurkandeniz@gmail.com (M.F.D.); irfan.sahin@sbu.edu.tr (I.S.); 2 Department of Cardiology, Kartal Kosuyolu Training and Research Hospital, Istanbul 34865, Turkey; kamilgulsen2000@yahoo.com; 3 Department of Cardiology, Konya Farabi Hospital, Konya 42250, Turkey; muziyrek@yahoo.com; 4 Department of Cardiology, Biruni University Faculty of Medicine, Istanbul 34295, Turkey; dremrahozdemir@gmail.com; 5 Department of Cardiology, Istanbul Medipol Universitesi Uluslararasi Tip Fakultesi, Istanbul 34214, Turkey; dreokuyan@hotmail.com

**Keywords:** transcatheter aortic valve replacement, V1-R wave amplitude, systolic pulmonary artery pressure, mortality

## Abstract

*Background and Objectives*: The function of the right heart and the pulmonary artery pressure have been linked to outcomes following transcatheter aortic valve replacement (TAVR). This study utilized the V1-R wave amplitude from pre-procedural electrocardiography as an indicator of pulmonary artery pressure, examining its connection to one-year mortality post-TAVR. *Materials and Methods*: This retrospective study, conducted at a single center, involved patients with severe symptomatic aortic valve stenosis who underwent TAVR between March 2014 and September 2023. The study analyzed patients’ pre-procedural V1-R wave amplitude on electrocardiography and systolic pulmonary artery pressure measured by transthoracic echocardiography. Patients who died within one year were classified as non-survivors; others as survivors. *Results*: Of the 236 patients, 56 (23.7%) died within one year of follow-up. A cut-off value of 66.5 µV for V1-R wave amplitude was associated with 66.1% sensitivity and 66.7% specificity (AUC: 0.735; 95% CI: 0.660–0.811), and 55.5 mmHg for systolic pulmonary artery pressure (sPAP) was associated with 67.9% sensitivity and 68.3% specificity (AUC: 0.708; 95% CI: 0.624–0.793) in predicting one-year mortality. The independent predictors of increased mortality included a history of cerebrovascular disease, elevated sPAP, and a high R-wave amplitude in lead V1. In contrast, statin use emerged as an independent predictor of reduced mortality. *Conclusions*: The V1-R wave amplitude and sPAP measured before TAVR were independent predictors of mortality following TAVR, highlighting the significance of right heart function.

## 1. Introduction

Aortic valve stenosis (AS) is the most common valvular disease, affecting approximately 2–4% of the elderly population [1]. Medical treatment for AS has not been shown to improve patient outcomes; thus, surgical aortic valve replacement or transcatheter aortic valve replacement (TAVR) are the primary treatment options [2]. Despite advancements in technique, procedure-related complications remain common and are associated with significant morbidity and mortality [3].

Electrocardiography (ECG) plays a crucial role in the diagnosis and management of cardiovascular diseases. It is a simple, cost-effective, and non-invasive tool that provides valuable diagnostic information. The initial phase of ventricular activation, known as septal stimulation, represents left-to-right depolarization of the septum and results in a small positive deflection (R-wave) in lead V1. Recent study show that a higher amplitude of the V1-R wave is linked to an elevated mean pulmonary artery pressure, which properly reflects right ventricular overload as measured by right heart catheterization in individuals with idiopathic pulmonary arterial hypertension (PAH) [4]. Additionally, a decrease in R-wave amplitude in lead V1 by ≥1 mm after three months of treatment in critically ill patients with idiopathic or heritable PAH was associated with improved survival outcomes [5]. The presence of myocardial scars in severe AS patients detected by cardiac magnetic resonance imaging were shown to be independently associated with mortality [6]. Patients diagnosed with left ventricular non-ischemic cardiomyopathy (NICM) commonly experience myocardial peri-mitral annular fibrosis, particularly characterized by low bipolar voltage areas located in a basal–lateral distribution, which aligns with scarring. An R-wave amplitude of ≥0.15 mV recorded in lead V1 has been identified as an indicator of left ventricular scarring in the basal–lateral regions, which typically forms the substrate for the onset of reentrant ventricular tachycardia (VT) in patients suffering from NICM [7].

TAVR improves both survival and quality of life in individuals with severe symptomatic aortic stenosis compared to medical treatment; however, the mortality rates after TAVR are still high. Elderly patients with various comorbidities are candidates for TAVR, and the impact of longstanding pressure overload as a result of severe AS on the myocardium and other heart valves also alters the prognosis. The presence of pulmonary hypertension (PHT) has been shown to be a marker of poor prognosis after aortic valve replacement. The development of PHT in individuals with aortic stenosis is complex, primarily driven by the association between elevated left ventricular filling pressure and pulmonary artery pressure [8,9]. In our study, the V1-R wave amplitude on pre-procedural ECG was used as an indicator of pulmonary artery pressure, and the relationship between V1-R wave amplitude and 1-year mortality after TAVR was evaluated.

## 2. Materials and Methods

### 2.1. Study Population

Patients older than 18 years with severe symptomatic AS who underwent TAVR between March 2014 and September 2023 were included in this retrospective, single-center study. Demographic, clinical, laboratory, and procedural data were obtained from hospital records and electronic databases. The risk assessment of patients was performed using the Society of Thoracic Surgeons mortality risk score, with all participants classified as intermediate-to-high risk [10]. All patients were evaluated with transthoracic echocardiography (Vivid S70; GE Medical System, Horten, Norway) and contrast-enhanced computed tomography. Additional assessments, including cardiac catheterization and/or transesophageal echocardiography, were conducted when necessary. Patients who died during the index hospitalization, had a left bundle branch block or a right bundle branch block, or with missing clinical, laboratory, electrocardiographic, or echocardiographic data were excluded from the study. Cerebrovascular accident (CVA) [11], coronary artery disease [12], hypertension [13], diabetes mellitus [14], atrial fibrillation [15], chronic kidney disease [16], hyperlipidemia [17], peripheral artery disease [18], and chronic obstructive pulmonary disease [19] were described according to established definitions.

Patients who were deceased within one year of follow-up formed the non-survivors, whereas the remaining formed the survivors group. The patients were further categorized according to V1-R wave amplitude as high and low V1-R and systolic pulmonary artery pressure (sPAP) as high and low sPAP.

The study was approved by the local Clinical Trials Ethics Committee of the hospital and conducted in accordance with the principles of the Declaration of Helsinki.

### 2.2. TAVR Procedure

All patients with symptomatic severe AS underwent comprehensive evaluation via transthoracic echocardiography to assess the valve morphology, disease severity, cardiac function, and aortic valve calcification. Additionally, contrast-enhanced computed tomography was used to evaluate the anatomy of the aortic valve, aortic annulus, and aorta, as well as to assess the peripheral vasculature and coronary ostium–annulus distance. Patient eligibility for TAVR was determined by a multidisciplinary heart team.

All procedures were performed by an experienced team using a transfemoral approach with local anesthesia and conscious sedation. Alternative access routes, such as the subclavian or carotid artery, and transapical access were used in cases where the transfemoral approach was not feasible. Balloon-expandable or self-expandable valves were selected based on aortic valve morphology and root anatomy.

### 2.3. Electrocardiography

A standard 12-lead ECG using an ECG-1350K (Nihon Kohden, Tokyo, Japan) was recorded in the supine position at a paper speed of 25 mm/s and a sensitivity of 1 mV = 10 mm. Electrocardiographic measurements were analyzed using the National Institutes of Health (NIH) ImageJ 1.54p software by a cardiologist blinded to the patients’ clinical data. The R-wave amplitudes were recorded in microvolts from the isoelectric PR segment to the top of the R-waves in leads V1 and aVR; a minimum of two amplitudes were recorded and averaged for each assessment. Randomly selected 10 patients were evaluated for intra- and inter-observer variability.

### 2.4. Statistical Analysis

Statistical analyses were performed using SPSS software version 26 (IBM, Armonk, NY, USA). Categorical variables were expressed as numbers and percentages. The Kolmogorov–Smirnov test was used to assess the normality of continuous variables, which were reported as mean ± standard deviation or median with interquartile range.

Comparisons between categorical variables were conducted using the Pearson Chi-square test or Chi-square test with Yates’ continuity correction, when appropriate. Continuous variables were compared using either the Mann–Whitney U-test or Student’s *t*-test, depending on the data distribution. Receiver operating characteristic curve analysis was conducted to assess the predictive value of the V1-R wave amplitude and sPAP for one-year mortality. We used Cox regression analysis to determine the association between independent variables and mortality. Variables that were statistically significant in the univariable analysis were included in the multivariable Cox regression model. The Kaplan–Meier test was used to estimate survival rates, and the log-rank test was used for group comparisons. Statistical significance was set at a two-tailed *p*-value < 0.05.

## 3. Results

**Figure 1:**
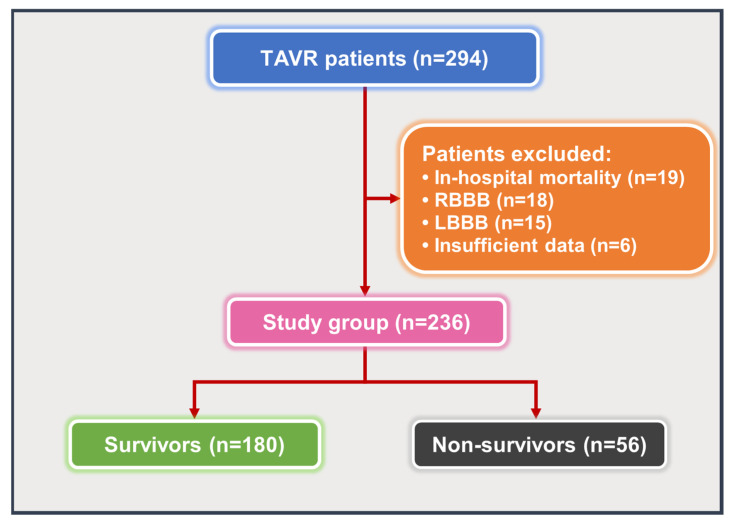
Flowchart of the study. Abbreviations: LBBB: left bundle branch block; RBBB: right bundle branch block; TAVR: transcatheter aortic valve replacement.

We initially evaluated 294 patients with severe symptomatic AS who underwent TAVR. After applying the exclusion criteria, 236 patients were included in the final analysis (Figure 1). The mean age was 78.34 ± 6.81 years, and 54.2% of the patients were female. One-year mortality occurred in 56 (23.7%) patients.

The ECGs and measurements of randomly selected 10 patients were reanalyzed by the same observer (OI) to assess intra-observer variability. For inter-observer variability, the same patients and same images were analyzed by a second observer (ED). The Pearson correlation co-efficient for V1-R wave amplitude was 0.88 for intra-observer and 0.86 for inter-observer variability.

There were no significant differences between survivors and non-survivors in terms of age, sex, body mass index, Society of Thoracic Surgeons mortality risk score, history of chronic kidney disease, coronary artery disease, diabetes mellitus, hypertension, peripheral artery disease, chronic obstructive pulmonary disease, atrial fibrillation, implanted valve type, contrast volume, hemoglobin levels, ejection fraction, mean aortic valve gradient, QRS duration, aVR-R wave amplitude, and medical treatment (use of anticoagulants, antiplatelet agents, or renin–angiotensin system inhibitors). The CVA (7.8% vs. 19.6%; *p* = 0.012), sPAP (51.6 ± 9.9 vs. 59.2 ± 11.5; *p* < 0.001), and amplitude of the V1-R wave [44 (25–79.5) vs. 99 (54–148); *p* < 0.001] were significantly higher in the non-survivors group. Nevertheless, the incidence of hyperlipidemia (71.1% vs. 53.6%; *p* = 0.015) and the use of beta-blockers (66.7% vs. 51.8%; *p* = 0.044) and statins (48.3% vs. 28.6%; *p* = 0.009) was significantly lower in the non-survivors group (Table 1).

**Figure 2:**
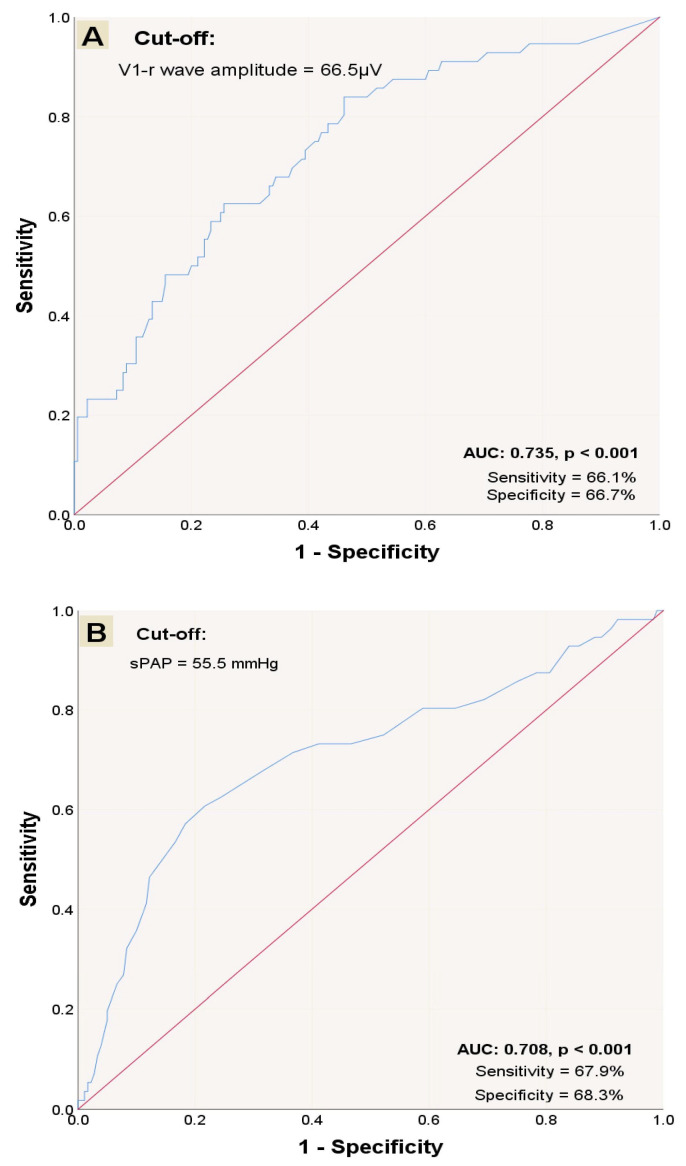
ROC curves’ performance for diagnosing one-year mortality; diagnostic accuracy of V1-R wave amplitude (**A**) and sPAP (**B**). Abbreviations: AUC: area under the curve; ROC: receiver operating characteristic. The red diagonal line at a 45° angle acts as the reference line, as it represents the ROC curve for random classification. The blue ROC curve is generated by plotting the true positive rate against the false positive rate for the corresponding variable.

Receiver operating characteristic curve analysis was performed to evaluate the diagnostic accuracy of V1-R wave amplitude and sPAP. A cut-off value of 66.5 µV for V1-R wave amplitude was associated with 66.1% sensitivity and 66.7% specificity (AUC: 0.735; 95% CI: 0.660–0.811) (Figure 2A), and 55.5 mmHg for sPAP was associated with 67.9% sensitivity and 68.3% specificity (AUC: 0.708; 95% CI: 0.624–0.793) (Figure 2B) in prediction of mortality within one year. Based on the established cut-offs, 97 patients were classified into the high V1-R group and 95 patients were categorized into the high sPAP group. Two groups were formed based on the V1-R wave amplitude: high V1-R and low V1-R. Hypertension was significantly lower, whereas sPAP was significantly higher in the high V1-R group (Table 2).

Univariable and multivariable Cox regression analyses were conducted to further investigate the individual risk factors linked to mortality within one year. A positive association was found between CVA, sPAP, and elevated V1-R and aVR-R wave amplitude, whereas statin use was negatively associated with mortality in the univariable Cox regression analysis. A multivariate Cox regression model was used to assess these factors. The analysis indicated that CVA (HR 2.336; 95% CI 1.195–4.566; *p* = 0.013), high sPAP (HR 2.920; 95% CI 1.639–5.202; *p* < 0.001), and high V1-R wave amplitude (HR 2.376; 95% CI 1.327–4.254; *p* = 0.004) were identified as independent predictors of increased mortality, whereas statin use (HR 0.482; 95% CI 0.270–0.861; *p* = 0.013) was an independent predictor of lower mortality rates within one year after TAVR (Table 3).

**Figure 3:**
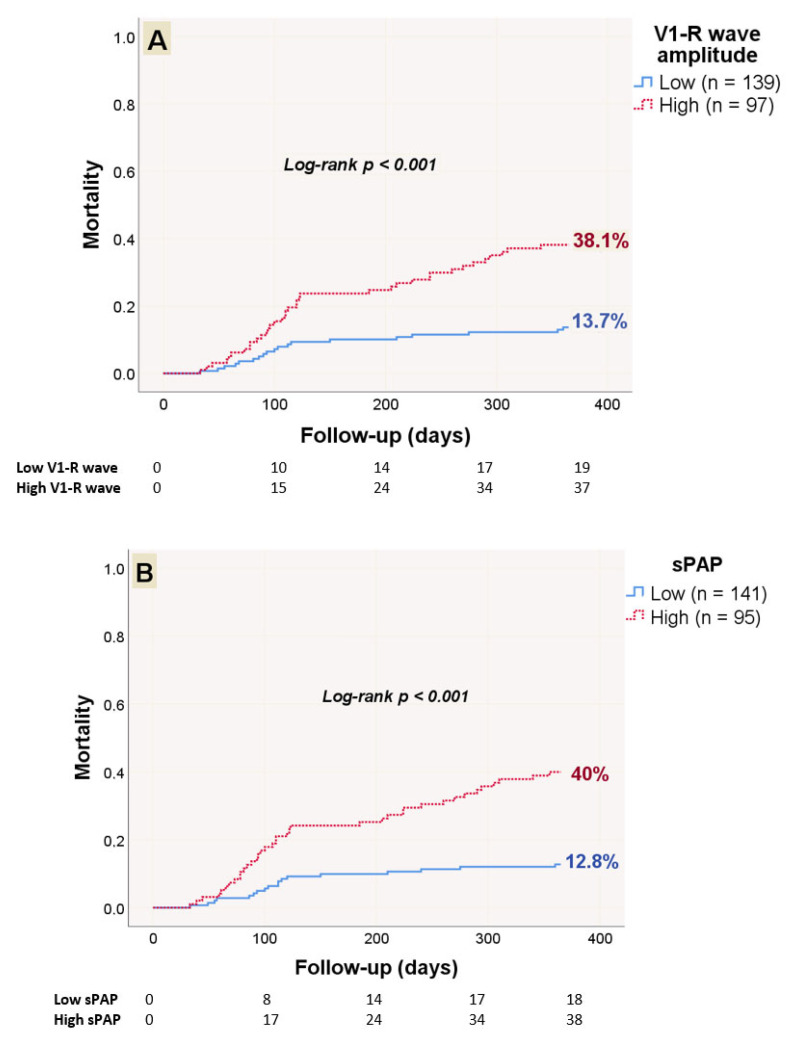
Kaplan–Meier analyses of mortality over the course of one year regarding low and high V1-R groups (**A**) and low and high sPAP groups (**B**).

The Kaplan–Meier estimate indicated that the mortality rate over 12 months was 13.7% for the low V1-R group and 38.1% for the high V1-R group (log-rank *p* < 0.001) (Figure 3A). Furthermore, the mortality rate was 12.8% in the low sPAP group and 40% in the high sPAP group (log-rank *p* < 0.001) (Figure 3B).

## 4. Discussion

A high V1-R wave amplitude on pre-procedural ECG, sPAP, and a history of CVA were independent predictors of increased mortality, whereas statin use was an independent predictor of reduced mortality after successful TAVR within one year of follow-up. The results indicate that assessing the ECG before TAVR, especially by measuring the V1-R wave amplitude, could offer further prognostic information for evaluating the risk in patients undergoing TAVR. Elevated sPAP along with increased V1-R amplitude may reflect the role of right heart functions on TAVR mortality.

The presence of PHT was detected as a marker of poor prognosis after surgical or transcatheter aortic valve replacement [20,21,22,23,24]. A positive correlation has been reported between the V1-R wave amplitude and both right ventricular diameter and mean pulmonary artery pressure, as assessed by right heart catheterization in patients with idiopathic PAH [4]. Additionally, a significant reduction in V1-R wave amplitude was observed following pulmonary endarterectomy in patients with chronic thromboembolic PAH, which was associated with improved right ventricular function [25]. In critically ill patients with idiopathic or heritable PAH, a decrease in V1-R amplitude by ≥1 mm after three months of targeted therapy was found to correlate with improved survival outcomes [5]. These findings suggest that an increased V1-R amplitude reflects right ventricular pressure overload and hypertrophy.

In the present study, we examined the relationship between pre-TAVR sPAP and V1-R wave amplitude. We found that sPAP was significantly higher in patients with elevated V1-R amplitudes, indicating a greater degree of right ventricular load, which may contribute to increased mortality risk. Although prior investigations focused on patients with PAH, our findings demonstrate that elevated baseline V1-R wave amplitude is also associated with increased mortality in patients undergoing TAVR.

Furthermore, myocardial scarring detected by cardiac magnetic resonance imaging in patients with severe AS has been independently associated with mortality [6]. In patients with left ventricular NICM, peri-mitral annular fibrosis, particularly in the basal–lateral regions, often presents as areas of low bipolar voltage and serves as a substrate for reentrant ventricular tachycardia. Notably, an R-wave amplitude ≥ 0.15 mV in lead V1 has been proposed as a non-invasive marker of left ventricular scar in this distribution [7]. In our study, a high V1-R amplitude was identified as an independent predictor of one-year mortality. Although arrhythmic death was not specifically investigated, it may have contributed to the observed increase in mortality.

Elevated baseline sPAP exceeding 60 mmHg was identified as an independent predictor of 1-year mortality after TAVR by Testa et al. [26] in previous research. Similarly, the sPAP assessed by transthoracic echocardiography was significantly higher in the non-survivors group in our study, and a cut-off value of 55.5 mmHg for sPAP was associated with 67.9% sensitivity and 68.3% specificity for predict one-year mortality.

The relationship between V1-R wave amplitude and adverse outcomes has been explored in various cardiac conditions, and high baseline values have been linked to worse prognosis in patients with PAH and non-ischemic cardiomyopathy [4,5,7,25,27]. This study is the first to evaluate the association between V1-R wave amplitude and mortality after TAVR. The potential relationship between high V1-R wave amplitude and impaired right heart function or malignant arrhythmias could contribute to increased mortality. Elevated sPAP in the non-survivors group, along with high V1-R wave amplitude levels, may indicate impaired right heart function, which could be linked to higher mortality rates in our study population. These findings highlight the need for further prospective studies to confirm our results.

A study revealed that the long-term mortality rate was significantly lower in the hyperlipidemia group following surgical aortic valve replacement [28]. Reduction of midterm mortality with statin therapy following TAVR was demonstrated in a meta-analysis [29]. In our study, 65.2% of patients were receiving statin treatment, and those patients had a significantly lower mortality rate. The decreased mortality in the hyperlipidemia group was thought to be due to statin use. This mortality benefit may be attributed to the anti-inflammatory, antithrombotic, and antioxidant effects of statins. Additionally, the patients on statin therapy might have received guideline-directed medical therapy and have higher compliance.

### Limitations

Our study has certain limitations, including its retrospective, single-center design and relatively small sample size with a short-term follow-up. Only patients with complete datasets were included in the analysis, whereas those with missing information were excluded, resulting in our study not being a consecutive series of patients. Advanced evaluation of right heart function, including tricuspid annular plane systolic excursion, peak systolic myocardial velocity using tissue Doppler imaging, fractional right ventricular area change, and right ventricular strain might provide further prognostic information. Conduction disturbances frequently occur in patients with AS. Patients who had existing rhythm disturbances and bundle branch blocks were not included. Consequently, this could affect how broadly our findings can be applied. The effect of TAVR on V1-R amplitude could provide additional information; however, the frequent occurrence of bundle branch block, atrioventricular block, or paced rhythm following the procedure makes it difficult to assess the V1-R amplitude in these situations. A cut-off value was defined however prospective validations would strengthen findings in larger multicentric cohorts. Since TAVI is getting more common, the device technology was improved during years. Our study population consist of patients between 2014 and 2023 and the developments in device and implantation techniques and operators’ experience might have impact on results. Despite these limitations, our findings provide insights into a clinically relevant association that warrants further investigation.

## 5. Conclusions

In this study, we demonstrated that a higher V1-R wave amplitude measured on pre-procedural ECG and sPAP had effective discriminatory power in determining one-year mortality following TAVR. Our results indicate that evaluating ECG records prior to TAVR, especially assessing the V1-R wave amplitude, could offer valuable prognostic information for risk assessment in TAVR candidates. Additionally, increased sPAP combined with higher V1-R wave amplitude may indicate the significance of right heart function in mortality related to TAVR. As a result, future studies are needed to clarify the potential role of sPAP and pre-procedural V1-R wave amplitude in the prognosis of patients with severe AS undergoing TAVR.

## Figures and Tables

**Table 1:** Baseline characteristics of patients in terms of mortality.

Variables	Total Population (*n* = 236)	Survivors (*n* = 180)	Non-Survivors (*n* = 56)	*p*-Value
Age, (years)	78.34 ± 6.81	77.87 ± 6.75	79.88 ± 6.83	0.054
Female sex, n (%)	128 (54.2)	100 (55.6)	28 (50)	0.466
BMI, (kg/m^2^)	27.98 ± 4.29	28.11 ± 4.41	27.54 ± 3.86	0.397
STS score	7.46 [6.16–10.3]	7.3 [5.83–10]	8.24 [6.5–11.75]	0.091
CKD grade ≥ 3, n (%)	116 (49.2)	84 (46.7)	32 (57.1)	0.171
CAD, n (%)	165 (69.9)	123 (68.3)	42 (75.0)	0.342
DM, n (%)	108 (45.8)	83 (46.1)	25 (44.6)	0.847
Hypertension, n (%)	179 (75.8)	139 (77.2)	40 (71.4)	0.376
CVA, n (%)	25 (10.6)	14 (7.8)	11 (19.6)	0.012
PAD, n (%)	66 (28)	46 (25.6)	20 (35.7)	0.139
COPD, n (%)	117 (49.6)	88 (48.9)	29 (51.8)	0.705
Hyperlipidemia, n (%)	158 (66.9)	128 (71.1)	30 (53.6)	0.015
AF, n (%)	58 (24.6)	40 (22.2)	18 (32.1)	0.132
AC, n (%)	56 (23.7)	38 (21.1)	18 (32.1)	0.090
Antiplatelet, n (%)	163 (69.1)	129(71.7)	34 (60.7)	0.121
BB usage, n (%)	149 (63.1)	120 (66.7)	29 (51.8)	0.044
RAS inhibitor usage, n (%)	159 (67.4)	124 (68.9)	35 (62.5)	0.373
Statin usage, n (%)	103 (43.6)	87 (48.3)	16 (28.6)	0.009
SE valve, n (%)	171 (72.5)	134 (74.4)	37 (66.1)	0.221
Contrast, (mL)	244.72 ± 76.28	239.42 ± 75.43	261.79 ± 77.19	0.055
Hemoglobin, (gr/dL)	11.46 ± 1.88	11.57 ± 1.8	11.11 ± 2.09	0.112
EF (%)	60 [50–60]	60 [50–60]	55 [45–60]	0.186
Mean AVG, (mmHg)	49.21 ± 9.95	49.38 ± 10.27	48.66 ± 8.9	0.637
sPAP, (mmHg)	53.4 ± 10.8	51.6 ± 9.9	59.2 ± 11.5	<0.001
V1-R wave amplitude, (µV)	53 [30–104.5]	44 [25–79.5]	99 (54–148]	<0.001
aVR-R wave amplitude, (µV)	46 [22–80.75]	45 [21.25- 76]	50 [23.75–107]	0.217
QRS wide, (ms)	95.08 ± 12.9	95.48 ± 12.94	93.80 ± 12.78	0.396

Abbreviations: AC: anticoagulant; AF: atrial fibrillation; AVG: aortic valve gradient; BB: beta-blocker; BMI: body mass index; CAD: coronary artery disease; CKD: chronic kidney disease; COPD: chronic obstructive pulmonary disease; CVA: cerebrovascular accident; DM: diabetes mellitus; EF: ejection fraction; PAD: peripheral artery disease; RAS: renin–angiotensin system; SE: self-expandable; sPAP: systolic pulmonary artery pressure; STS: The Society of Thoracic Surgeons. *p*-value < 0.05 was regarded as statistically significant.

**Table 2:** Baseline characteristics of patients grouped by V1-R wave amplitude.

Variables	Low V1-R (*n* = 139)	High V1-R (*n* = 97)	*p*-Value
Age, (years)	78.6 ± 6.6	78 ± 7.1	0.519
Female sex, n (%)	80 (57.6)	48 (49.5)	0.221
BMI, (kg/m^2^)	28 ± 4.2	27.9 ± 4.4	0.845
STS score	7.6 [6.3–10.4]	7.3 [5.8–10.3]	0.464
CKD grade ≥ 3, n (%)	70 (50.4)	46 (47.4)	0.657
CAD, n (%)	100 (71.9)	65 (67)	0.416
DM, n (%)	65 (46.8)	43 (44.3)	0.712
Hypertension, n (%)	115 (82.7)	64 (66)	0.003
CVA, n (%)	12 (8.6)	13 (13.4)	0.241
PAD, n (%)	44 (31.7)	22 (22.7)	0.131
COPD, n (%)	73 (52.5)	44 (45.4)	0.279
Hyperlipidemia, n (%)	93 (66.9)	64 (66)	0.882
AF, n (%)	36 (25.9)	22 (22.7)	0.572
AC, n (%)	34 (24.5)	22 (22.7)	0.752
Antiplatelet, n (%)	96 (69.1)	67 (69.1)	0.999
BB usage, n (%)	91 (65.5)	58 (59.8)	0.374
RAS inhibitor usage, n (%)	100 (71.9)	59 (60.8)	0.073
Statin usage, n (%)	58 (41.7)	45 (46.4)	0.477
SE valve, n (%)	104 (74.8)	67 (69.1)	0.331
Contrast, (mL)	244.6 ± 78.2	244.9 ± 73.9	0.970
Hemoglobin, (gr/dL)	11.47 ± 2	11.45 ± 1.7	0.940
EF (%)	60 [48–60]	60 [50–60]	0.684
Mean AVG, (mmHg)	48.9 ± 10.4	49.6 ± 9.22	0.594
sPAP, (mmHg)	51.6 ± 10	55.9 ± 11.5	0.003
QRS width, (ms)	95.3 ± 12.7	94.7 ± 13.2	0.738

Abbreviations: AC: anticoagulant; AF: atrial fibrillation; AVG: aortic valve gradient; BB: beta-blocker; BMI: body mass index; CAD: coronary artery disease; CKD: chronic kidney disease; COPD: chronic obstructive pulmonary disease; CVA: cerebrovascular accident; DM: diabetes mellitus; EF: ejection fraction; PAD: peripheral artery disease; RAS: renin–angiotensin system; SE: self-expandable; sPAP: systolic pulmonary artery pressure; STS: The Society of Thoracic Surgeons. *p*-value < 0.05 was regarded as statistically significant.

**Table 3:** Predictors of one-year survival in transcatheter aortic valve replacement patients.

Variables	Univariable HR (95% CI)	*p*-Value	Multivariable HR (95% CI)	*p*-Value
Age	1.040 (0.999–1.082)	0.056		
Female sex	0.821 (0.486–1.387)	0.461		
BMI	0.976 (0.917–1.039)	0.446		
STS score	1.045 (0.992–1.100)	0.100		
CKD grade ≥ 3	1.455 (0.857–2.470)	0.165		
CAD	1.359 (0.742–2.489)	0.320		
Diabetes mellitus	0.989 (0.584–1.676)	0.969		
Hypertension	0.737 (0.375–1.450)	0.377		
CVA	2.483 (1.283–4.805)	0.007	2.336 (1.195–4.566)	0.013
PAD	1.509 (0.873–2.606)	0.140		
COPD	1.068 (0.632–1.803)	0.807		
Atrial fibrillation	1.484 (0.847–2.600)	0.168		
Contrast volume	1.003 (1.000–1.006)	0.067		
Self-expandable valve	0.708 (0.407–1.231)	0.221		
LV-EF	0.985 (0.962–1.008)	0.193		
AV mean gradient	0.993 (0.968–1.020)	0.616		
Baseline hemoglobin	0.887 (0.765–1.028)	0.112		
High sPAP	3.645 (2.079–6.391)	<0.001	2.920 (1.639–5.202)	<0.001
Hyperlipidemia	0.539 (0.319–0.911)	0.021		
Statin usage	0.482 (0.270–0.861)	0.014	0.478(0.266–0.856)	0.013
Beta-blocker usage	0.600 (0.355–1.014)	0.056		
High V1-R amplitude	3.180 (1.828–5.533)	<0.001	2.376 (1.327–4.254)	0.004
QRS wide	0.992 (0.971–1.012)	0.429		
aVR-R amplitude	1.003 (1.0003–1.007)	0.034	1.001 (0.998–1.005)	0.479

Abbreviations: AV: aortic valve; BMI: body mass index; CAD: coronary artery disease; CKD: chronic kidney disease; COPD: chronic obstructive pulmonary disease; CVA: cerebrovascular accident; EF: ejection fraction; LV: left ventricle; PAD: peripheral artery disease; sPAP: systolic pulmonary artery pressure; STS: The Society of Thoracic Surgeons. *p*-value < 0.05 was regarded as statistically significant.

## Data Availability

The datasets utilized and/or examined during this study can be obtained from the corresponding author with a reasonable request.

## Abbreviations

The following abbreviations are used in this manuscript:

AS	Aortic valve stenosis
CVA	Cerebrovascular accident
ECG	Electrocardiography
PAH	Pulmonary arterial hypertension
PHT	Pulmonary hypertension
sPAP	Systolic pulmonary artery pressure
TAVR	Transcatheter aortic valve replacement
